# Study the Effect of Intraperitoneal Dexamethasone, Dexmedetomidine, and Their Combination on PONV After Laparoscopic Cholecystectomy: A Randomized Triple-Blind Trial

**DOI:** 10.1155/anrp/4976637

**Published:** 2025-02-24

**Authors:** Hany Bauiomy, Neveen A. Kohaf, Mohammed Saad, Zaky Ftouh Rashed, Ahmed M. Abosakaya

**Affiliations:** ^1^Department of Anesthesia & Intensive Care, Faculty of Medicine, Benha University, Benha, Egypt; ^2^Department of Clinical Pharmacy, Faculty of Pharmacy (Girls), Al-Azhar University, Cairo, Egypt; ^3^Department of Anesthesia, Intensive Care and Pain Management, Faculty of Medicine, Al-Azhar University, Cairo, Egypt

**Keywords:** cholecystectomy, dexamethasone, dexmedetomidine, intraperitoneal, PONV

## Abstract

**Background:** Postoperative nausea and vomiting (PONV) are major adverse consequences following laparoscopic cholecystectomy. Several drugs have been used to combat its occurrence.

**Objective:** This study aimed to show the efficacy of the intraperitoneal route and compare different antiemetic effects of dexamethasone, dexmedetomidine, and their combination on PONV after laparoscopic cholecystectomy under general anesthesia in a tertiary care hospital.

**Design:** Prospective randomized triple-blind study.

**Setting:** The trial was conducted at Benha University Hospitals. The trial was done from August 2023 to April 2024.

**Patients:** Two hundred and forty patients aged 20–50 years, Apfel Score 1, the American Society of Anesthesiologists (ASA) physical status Classification I or II who underwent laparoscopic cholecystectomy. Exclusion criteria were a history of psychotic illnesses, Parkinson's disease, motion disorder, and a history of chemotherapy.

**Interventions:** Patients were randomized equally into four groups. Group I (control group) received 20 mL normal saline, Group II (dexamethasone group) received 8 mg dexamethasone, Group III (dexmedetomidine group) received dexmedetomidine 1mic/kg, and Group IV (combination group) received the combination of both dexamethasone (8 mg) + dexmedetomidine (1mic/kg). The medications were diluted in 20 mL normal saline.

**Main Outcome Measures:** The incidence of PONV encountered by patients in the first 24 h following surgery was recorded.

**Results:** Nausea was reported in 26 (43.33%), 10 (16.67%), 11 (18.33%), and 6 (10%) in Groups I, II, III, and IV, respectively. Vomiting was observed in 25 (41.67%), 11 (18.33%), 10 (16.67%), and five (8.33%) in Groups I, II, III, and IV, respectively. Antiemetic medication was required for 24 (40%), 11 (18.33%), 12 (20%), and eight (13.33%) in Groups I, II, III, and IV, respectively. Nausea, vomiting, and antiemetics requirements differed significantly among the four groups (*p* value < 0.05).

**Conclusions:** Intraperitoneal administration of dexamethasone and dexmedetomidine either alone or in combination decreased the incidence of PONV among patients scheduled for laparoscopic cholecystectomy.

**Trial Registration:** ClinicalTrials.gov identifier: NCT05988671

**Summary**

• Postoperative nausea and vomiting (PONV) are major adverse consequences following laparoscopic cholecystectomy.
• Intraperitoneal drug administration involves injecting medications directly into the peritoneal cavity.
• Intraperitoneal administration of dexamethasone and dexmedetomidine either alone or in combination decreased the incidence of PONV among patients scheduled for laparoscopic cholecystectomy.

## 1. Introduction

Laparoscopic cholecystectomy, a minimally invasive surgery for gallbladder removal, offers advantages such as reduced pain and shorter recovery times. However, PONV remains a common complication that can impact patient well-being [1, 2]. PONV, which is experienced by 20%–30% of surgical patients within 24 h postoperation, is attributed to anesthetic effects on the center for vomiting regulation in the medulla oblongata, or intraoperative hypoxia-induced nausea and vomiting [3].

PONV can lead to various complications and adverse events for patients, including pulmonary aspiration, airway obstruction, wound dehiscence, imbalances in electrolytes, dehydration, hypertension, flaps bleeding, stretching of sutures, late discharge, and increased risk of aspiration pneumonia [4]. Therefore, healthcare providers need to take preventive measures to minimize the occurrence of PONV and its associated complications.

PONV can be treated with various medications, such as blockers of dopamine and serotonin receptors, antihistamines, corticosteroids, anticholinergics, and sedatives [5]. The most commonly used drugs for relieving PONV are metoclopramide and droperidol [6, 7].

However, these agents can have potential side effects, including fatigue, agitation, amnesia for place and time, extrapyramidal symptoms, cardiovascular problems, hypotension, orthostatic hypotension, sleepiness, akathisia, increased liver enzymes, and agranulocytosis which limit their use in some patients [7].

Dexamethasone is a corticosteroid with anti-inflammatory properties and has been provided to reduce the incidence of PONV after surgery [8]. Dexmedetomidine is a potent agonist of α-2-adrenergic receptors with various properties, including sedative, anxiolytic, pain control, sympatholytic, and hemodynamic control [9]. A meta-analysis of randomized controlled trials revealed the antiemetic effect of dexmedetomidine [10].

While the intravenous (IV) route of administering medication being effective and rapid, it comes with several disadvantages. There is a higher risk of immediate systemic side effects and dosing errors [11]. Thus, searching for other routes is crucial.

Intraperitoneal drug administration involves injecting medications directly into the peritoneal cavity. Diffusion of drugs from the peritoneal cavity into surrounding tissues is a crucial consideration in this route of administration [12].

Intraperitoneal drug administration is gaining attention due to its potential benefits in managing postoperative pain and complications with fewer side effects when compared with other routes [13, 14]. Moreover, intraperitoneal administration has been compared to IV routes for its impact on PONV and provided a lower incidence of nausea, comparable incidence of vomiting, and severity of PONV with significantly lower side effects [13].

In the literature, only one study investigated the PONV reduction efficacy of the intraperitoneal route with dexamethasone, dexmedetomidine, and their combination after gynecological laparoscopic surgeries and concluded that combination therapy had a better effect than monotherapy [15]. To extrapolate this finding to another type of surgery, this triple-blind clinical trial study aimed to compare the prophylactic efficacy of dexamethasone and dexmedetomidine and their combination in reducing PONV in patients having laparoscopic cholecystectomy.

## 2. Methods

### 2.1. Ethics

Ethical approval for this study (Ethical Committee approval code: RC 10-3-2023) was provided by the Ethical Committee of Benha University Hospitals Ethical Committee, Benha, Egypt (Chairperson Prof Mahmoud Abd Elsabour) on 10 March 2023. And written informed consent was obtained from all subjects participating in the trial (date of registration: August 14, 2023).

This randomized prospective triple-blinded trial included 240 patients aged 20–50 years, Apfel Score 1, the American Society of Anesthesiologists (ASA) physical status Classification I or II who underwent laparoscopic cholecystectomy. The trial was done from August 2023 to April 2024. The trial was conducted at Benha University Hospitals.

Exclusion criteria were a history of psychotic illnesses, Parkinson's disease, motion disorder, patients with an Apfel score equal to or more than two, and a history of chemotherapy. The Apfel simplified risk score identifies the likelihood of PONV based on four critical factors: being female, a history of PONV or motion sickness, nonsmoking, and the utilization of opioids after surgery. According to the score, the frequencies of PONV corresponding to the accumulation of 0, 1, 2, 3, and 4 risk factors are approximately 10%, 20%, 40%, 60%, and 80%, in that order. Furthermore, patients are categorized into risk groups as “low,” “medium,” and “high,” based on having 0-1, 2-3, and four or more risk factors, respectively [16].

Nausea was described as an urge to vomit in the absence of expulsive muscle contractions. The definition of vomiting or emesis was the violent oral ejection of gastrointestinal components [17].

### 2.2. Randomization and Blindness

Computer‐generated randomization numbers were utilized to randomly assign 240 patients equally into four groups by permuted randomization.

Group I (control group) received 20 mL normal saline, Group II (dexamethasone group) received 8 mg dexamethasone, Group III (dexmedetomidine group) received dexmedetomidine 1mic/kg, and Group IV (combination group) received a combination of both dexamethasone + dexmedetomidine.

A nurse who was not involved in the study utilized opaque, sequentially numbered, and hermetically sealed envelopes to ensure a random assignment. The allocation ratio was 1:1:1:1 in a parallel manner. Patients, investigators, and outcome assessors were blinded to the experimental drugs. Another clinical pharmacist who did not participate in the trial's subsequent phases prepared the medications. All solution-containing syringes were identical in appearance.

### 2.3. Preoperative

Before the induction of anesthesia, an IV line was inserted, and all patients underwent routine monitoring and were connected to a monitor consisting of pulse oximetry, noninvasive blood pressure (BP), 5-lead ECG, a temperature probe, and capnography. All patients received 10 mg metoclopramide as preoperative antiemetic prophylaxis.

### 2.4. Intraoperative

The patients were subsequently transferred to the operating room to undergo surgery. All patients got the same method of general anesthesia: In the form of IV induction with 2 mg/kg propofol, fentanyl 2 mic/kg, and 0.5 mg/kg of atracurium improved intubation. Following intubation, a nasogastric tube was inserted to remove air and gastric contents from the stomach.

Isoflurane 1.5 MAC and atracurium 0.15 mg/kg every 20 min were used to maintain anesthesia. The pneumoperitoneum was used to keep intraabdominal pressure at 13–15 mmHg throughout the laparoscopic procedure. After the accomplishment of surgery, all study drugs were administered intraperitoneally (IP) by the surgeon as per group allocation before laparoscopic trocar withdrawal.

At the end of the operation, local anesthetic was injected at port site, and the neuromuscular blocker was neutralized using IV neostigmine 50 μg per kg and atropine 20 μg per kg.

Patients were given IV paracetamol 1 gm/8 h. If visual analog score (VAS) > 4 was observed, rescue analgesia ketorolac 30 mg 8 hourly was administered. Postoperative hemodynamics were recorded at PACU, 1, 2, 4, 8, 12, and 24 h.

The primary outcome was the incidence of nausea during the first 24 h, postoperatively. The secondary outcomes were the incidence of vomiting, the need for rescue antiemetic medicines (ondansetron 8 mg IV), and the recording of BP and heart rate (HR) during the first 24 h, postoperatively.

### 2.5. Sample Size Calculation

The sample size calculation was done by G∗Power 3.1.9.2 (Universitat Kiel, Germany). Based on the following considerations, 0.05 *α* error and 90% power of the study, allocation ratio 1:1:1:1, and five additional patients were provided to each group to combat dropout. Therefore, 240 patients were included.

### 2.6. Statistical Analysis

Statistical analysis was done by SPSS v28 (IBM, Chicago, IL, USA). Using the Shapiro–Wilk test and histograms, the normality of the data distribution was determined. Parametric quantitative data were given as mean and standard deviation (SD) and analyzed using the ANOVA (F) test with post hoc comparisons (Tukey). The Chi-square test was utilized to examine qualitative variables expressed as frequency and percentage (%). A two-tailed *p* value of 0.05 or less was judged statistically significant.

## 3. Results

**Figure 1:**
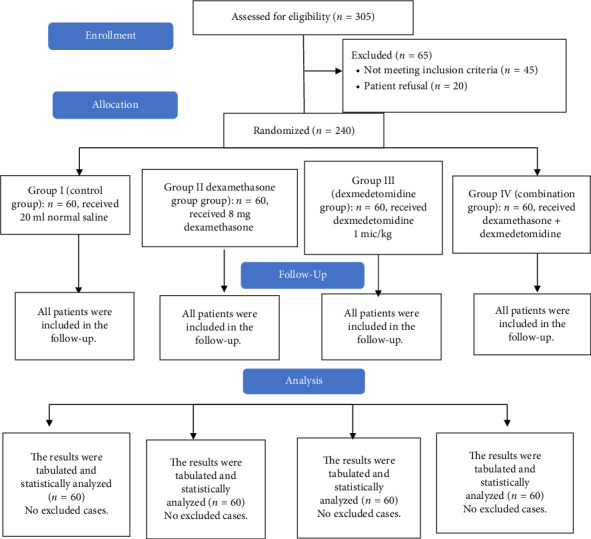
CONSORT flowchart of the enrolled patients.

In this study, eligibility was dedicated to 305 participants, 45 participants did not match the eligibility requirements, and 20 patients declined to contribute to the study. The remaining 240 patients were randomly assigned to four groups of equal size (60 patients per group). All allocated patients were monitored and statistically assessed (Figure 1).

Age, ASA physical status, sex, weight, height, BMI, and duration of surgery were matched among the four groups (Table 1).

Nausea was present in 26 (43.33%), 10 (16.67%), 11 (18.33%), and ix (10%) in Groups I, II, III, and IV, respectively. Vomiting occurred in 25 (41.67%), 11 (18.33%), 10 (16.67%), and five (8.33%) in Groups I, II, III, and IV, respectively. There were 24 (40%), 11 (18.33%), 12 (20%), and eight (13.33%) required antiemetic medication in Groups I, II, III, and IV, respectively. Nausea, vomiting, and antiemetics requirements were significantly different among the four groups (*p* value < 0.05). Post hoc analysis revealed significantly higher incidence of nausea, vomiting, and rescue of antiemetics in Group I compared to Group II, Group III, and Group IV (*p* < 0.05). While Group II showed an insignificant difference compared to Group III and IV (*p* > 0.05). Also Group III and IV showed comparable results (*p* > 0.05) (Table 2).

**Figure 2:**
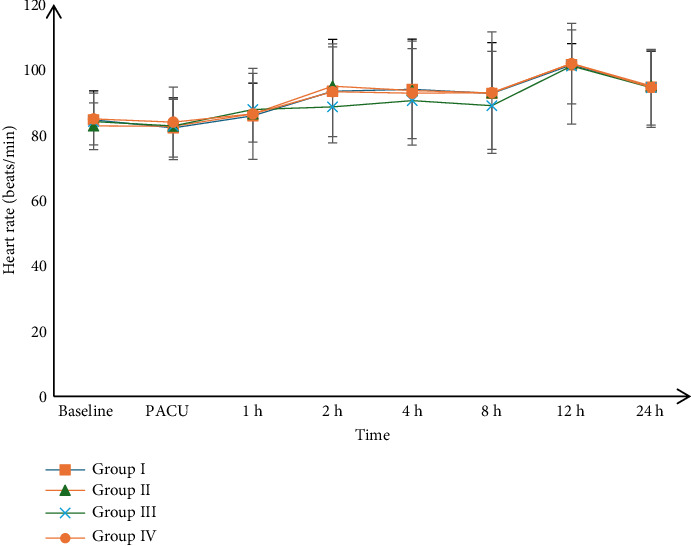
Postoperative heart rate (HR) among the studied groups.

**Figure 3:**
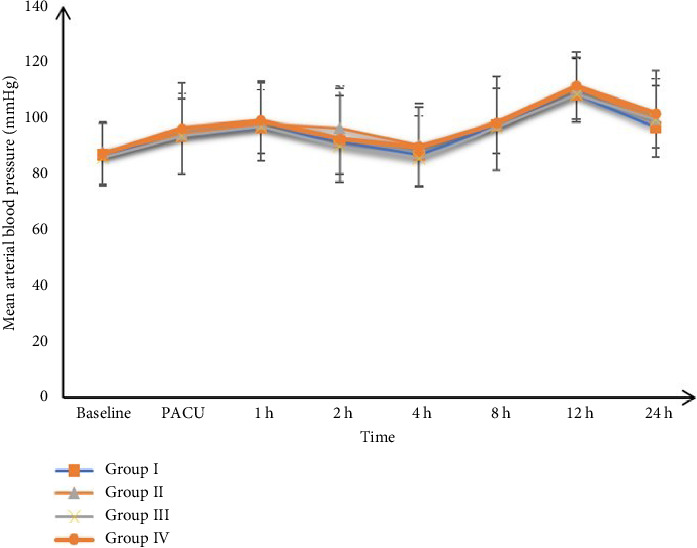
Postoperative mean blood pressure (MBP) among the studied groups.

Postoperative HR and mean BP measurements were insignificantly different among the four groups at PACU, 1, 2, 4, 8, 12, and 24 h, Figures 2 and 3.

## 4. Discussion

The purpose of this study was to compare the prophylactic efficacy of dexamethasone and dexmedetomidine and their combination in reducing PONV in patients having laparoscopic cholecystectomy. We found that intraperitoneal instillation of dexamethasone, dexmedetomidine, and a combination of both can significantly reduce the incidence of nausea and vomiting and rescue antiemetics compared to the control group in laparoscopic procedures. The effect of dexamethasone and dexmedetomidine could be mediated by their action on glucocorticoid receptors, prostaglandin, catecholamines, serotonin, and substance P. This reduces nociceptive stimulus during the acute phase of postoperative pain, decreasing the incidence of PONV [18].

We also found that intraperitoneal administration of combined dexamethasone and dexmedetomidine showed a lower incidence than monotherapy administration with no significant difference. The additive effect of this combination could explain this.

In addition, the follow-up of BP and HR measurements showed insignificant differences among the four groups, which is considered one of the intraperitoneal route's beneficial effects due to lower bioavailability.

Laparoscopic surgeries can increase the incidence of PONV through different mechanisms. Different types and doses of anesthetic agents can influence the likelihood of PONV [3]. The stress of surgery and the manipulation of the abdominal organs during laparoscopic procedures can stimulate the body's “vomiting reflex,” causing nausea and vomiting [19]. Carbon dioxide gas is always utilized during laparoscopic surgery to expand the abdominal cavity and create a workspace for the surgeon. The gas can irritate the diaphragm and peritoneum, potentially leading to postoperative discomfort and nausea [20]. Pneumoperitoneum pressure created by the insufflated gas in the abdominal cavity can affect the diaphragm and stimulate the vagus nerve, which can result in nausea and vomiting [21]. Increased surgical duration [22] and patient factors such as age, gender, a history of motion sickness, a history of PONV, and certain medical conditions can increase the risk of nausea and vomiting after surgery [3].

Numerous authors have studied the efficacy of intraperitoneal dexamethasone and dexmedetomidine in reducing pain and subsequently decreasing the incidence of PONV [23] after laparoscopic surgery [13, 24–26].

According to our results, nausea was present in 26 (43.33%), 10 (16.67%), 11 (18.33%), and six (10%) patients in Groups I, II, III, and IV, respectively. Vomiting occurred in 25 (41.67%), 11 (18.33%), 10 (16.67%), and five (8.33%) patients in Groups I, II, III, and IV, respectively. Antiemetic medication was required for 24 (40%), 11 (18.33%), 12 (20%), and eight (13.33%) patients in Groups I, II, III, and IV, respectively.

Compared to our study, Ismail et al. [13] compared the effect of intraperitoneal versus IV dexamethasone for reducing PONV after gynecological laparoscopic surgeries. Eighty patients were randomized equally to receive 8 mg dexamethasone intravenously (IV) or IP. Eleven women (27.5%) in the IV group versus only three (7.5%) women in the IP group experienced nausea during the first 24 h postlaparoscopy (*p* = 0.037). However, five patients (12.5%) in the IV group versus only two patients (5.0%) in the IP group experienced vomiting (*p* = 0.424). They reported that intraperitoneal dexamethasone at a dose of 8 mg at the end of gynecological laparoscopy reduces the incidence of postoperative nausea, which agreed with our findings but with a lower incidence of nausea and vomiting compared to our study that may be attributed to different surgeries.

Bakri et al. [27] equally randomized 86 adult patients scheduled for laparoscopic cholecystectomy to receive either a single IV dose of 1 μg/kg of dexmedetomidine or 8 mg dexamethasone before skin incision. They showed that compared to IV dexamethasone, IV dexmedetomidine lowers the severity and incidence rate of PONV without significant differences, which is comparable to our findings but takes a different route, which agrees with our study.

Thapa et al. [28] conducted a descriptive cross-sectional study among patients undergoing laparoscopic cholecystectomy under general anesthesia. Ondansetron 4 mg was given to all the patients as antiemetic prophylaxis around half an hour before the completion of surgery. Among 200 patients, PONV were seen in 28 (14%) (9.19–18.81, 95% confidence interval). Among them, seven (25%) of the patients experienced postoperative vomiting as well. Robles-Espinoza et al. [29] conducted an observational prospective study on 224 patients scheduled for elective laparoscopic cholecystectomy. The type of prophylactic medication that was received in most patients was an ondansetron. The prevalence of postoperative nausea was 33.03%, and vomiting was 31.25% in the first 24 h, which is lower than the prevalence in our population. Different anesthetic procedures, such as the use of nitrous gas, opioids, and periods of anesthesia, could account for the disparity.

Our results are also supported by Srivastava [15] who randomized one hundred ninety-two female patients undergoing laparoscopic hysterectomies into four groups. Group D1 received dexamethasone 8 mg, Group D2 received dexmedetomidine 1 μg/kg, Group D3 received dexamethasone 8 mg + dexmedetomidine 1 μg/kg combination, and Group D4 received 20 mL normal saline IP at the end of the procedure. The incidence of PONV within the first 24 h postoperatively was significantly decreased in the D1, D2, and D3 groups compared to Group D4 (*p*=0.001); however, there was no significant difference among patients in the D1, D2, and D3 groups.

According to our knowledge, no other studies have demonstrated the effects of intraperitoneal medication used for PONV prophylaxis on hemodynamic parameters.

The limitations of the current study are as follows. As the patients in our study were followed up to 24 h after surgery, the presence of nausea and vomiting after the observation period was not studied. Furthermore, because patients were lost to follow-up after 5 days, we were unable to assess the occurrence of long-term adverse effects of the studied medications. Because there are so few studies on the use of intraperitoneal dexamethasone and dexmedetomidine for the reduction of PONV in the literature, more research with different doses of these drugs and a combination of other postoperative antiemetic is needed to provide the best results in terms of PONV relief after laparoscopic surgeries.

## 5. Conclusions

Intraperitoneal administration of dexamethasone and dexmedetomidine either alone or in combination reduced the incidence of PONV among patients undergoing laparoscopic cholecystectomy.

## Figures and Tables

**Table 1:** Demographic data and duration of surgery of the studied groups.

	Group I (*n* = 60)	Group II (*n* = 60)	Group III (*n* = 60)	Group IV (*n* = 60)	*p* value
Age (years)	35.6 ± 10.02	35 ± 8.66	36.7 ± 8.39	34.9 ± 9.29	0.673

ASA	I	30 (50%)	31 (51.67%)	31 (51.67%)	0.988
	II	30 (50%)	29 (48.33%)	29 (48.33%)	

Sex	Male	32 (53.33%)	30 (50%)	31 (51.67%)	0.953
	Female	28 (46.67%)	30 (50%)	29 (48.33%)	

Weight (kg)	64.1 ± 4.14	63.2 ± 3.6	64.6 ± 3.03	63.4 ± 3.56	0.138

Height (cm)	163.5 ± 8.55	166.1 ± 8.76	166.2 ± 6.62	166.7 ± 7.7	0.117

BMI (kg/m^2^)	23.8 ± 2.68	23.1 ± 2.88	23.5 ± 2.14	22.9 ± 2.2	0.269

Duration of surgery (min)	66.4 ± 14.27	68.8 ± 12.21	69.6 ± 11.97	67.7 ± 12.66	0.558

*Note:* Data are presented as mean ± SD or frequency (%).

Abbreviations: ASA, American Society of Anesthesiologists; BMI, body mass index.

**Table 2:** Incidence of nausea, vomiting, and rescue of antiemetics among the studied groups.

		Group I (*n* = 60)	Group II (*n* = 60)	Group III (*n* = 60)	Group IV (*n* = 60)	*p* value	Post hoc
Nausea	Yes	26 (43.33%)	10 (16.67%)	11 (18.33%)	6 (10%)	< 0.001⁣^∗^	*P*1 = 0.0028⁣^∗^ *P*2 = 0.0056⁣^∗^ *P*3 < 0.0001⁣^∗^ *P*4 = 0.8101 *P*5 = 0.4205 *P*6 = 0.295
	No	34 (56.67%)	50 (83.33%)	49 (81.67%)	54 (90%)		

Vomiting	Yes	25 (41.67%)	11 (18.33%)	10 (16.67%)	5 (8.33%)	< 0.001⁣^∗^	*P*1 = 0.0096⁣^∗^ *P*2 = 0.0049⁣^∗^ *P*3 < 0.0001⁣^∗^ *P*4 = 0.8101 *P*5 = 0.1794 *P*6 = 0.2695
	No	35 (58.33%)	49 (81.67%)	50 (83.33%)	55 (91.67%)		

Rescue of antiemetics	Yes	24 (40%)	11 (18.33%)	12 (20%)	8 (13.33%)	0.003⁣^∗^	*P*1 = 0.0159⁣^∗^ *P*2 = 0.0284⁣^∗^ *P*3 = 0.002⁣^∗^ *P*4 = 0.8166 *P*5 = 0.617 *P*6 = 0.4624
	No	36 (60%)	49 (81.67%)	48 (80%)	52 (86.67%)		

*Note:* Data are presented as frequency (%). P1: comparing Group I to Group II, P2: comparing Group I to Group III, P3: comparing Group I to Group IV, P4: comparing Group II to Group III, P5: comparing Group II to Group IV, P6: comparing Group III to Group IV.

⁣^∗^Significant *p* value < 0.05.

## Data Availability

The datasets generated during and/or analyzed during the current study are available from the corresponding author upon reasonable request.
